# 16-[(*E*)-4-Bromo­benzyl­idene]-13-(4-bromo­phen­yl)-2-hy­droxy-11-methyl-1,11-diaza­penta­cyclo­[12.3.1.0^2,10^.0^3,8^.0^10,14^]octa­deca-3(8),4,6-triene-9,15-dione

**DOI:** 10.1107/S1600536810051585

**Published:** 2010-12-15

**Authors:** Raju Suresh Kumar, Hasnah Osman, Subbu Perumal, Jia Hao Goh, Hoong-Kun Fun

**Affiliations:** a School of Chemical Sciences, Universiti Sains Malaysia, 11800 USM, Penang, Malaysia; b Department of Organic Chemistry, School of Chemistry, Madurai Kamaraj University, Madurai 625 021, India; c X-ray Crystallography Unit, School of Physics, Universiti Sains Malaysia, 11800 USM, Penang, Malaysia

## Abstract

In the title pyrrolidine compound, C_30_H_24_Br_2_N_2_O_3_, the two fused pyrrolidine rings adopt envelope and twisted conformations, whereas the piperidine ring adopts an envelope conformation. The essentially planar 2,3-dihydro­indanone unit [maximum deviation = −0.0163 (19) Å] is inclined at inter­planar angles of 14.29 (9) and 61.07 (9)° to the two benzene rings. In the crystal, adjacent mol­ecules are linked into dimers by inter­molecular O—H⋯N and C—H⋯O hydrogen bonds. Short inter­molecular Br⋯Br inter­actions [3.5140 (6) Å] further inter­connect these dimers into double dimeric columns along the *b* axis.

## Related literature

For general background to and applications of the title pyrrolidine compound, see: Huryn *et al.* (1991 ▶); Suzuki *et al.* (1994 ▶); Waldmann (1995 ▶). For the preparation, see: Kumar *et al.* (2010**a* ▶,*b* ▶,c* ▶). For ring puckering analysis, see: Cremer & Pople (1975 ▶). For closely related pyrrolidine structures, see: Kumar *et al.* (2010**a* ▶,*b* ▶,c* ▶). For the stability of the temperature controller used in the data collection, see: Cosier & Glazer (1986 ▶).
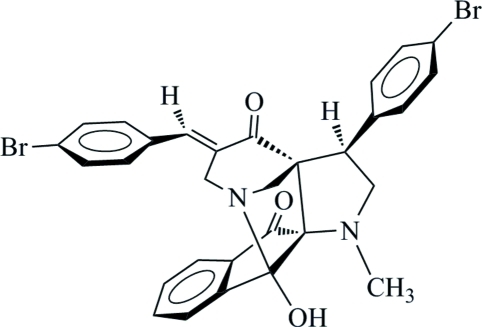


## Experimental

### 

#### Crystal data

C_30_H_24_Br_2_N_2_O_3_
*M* *_r_* = 620.33
Monoclinic, 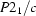

*a* = 13.3490 (18) Å
*b* = 9.1243 (12) Å
*c* = 22.541 (3) Å
β = 113.191 (6)°
*V* = 2523.7 (6) Å^3^
*Z* = 4
Mo *K*α radiation
μ = 3.25 mm^−1^
*T* = 100 K
0.39 × 0.38 × 0.18 mm

#### Data collection

Bruker APEXII DUO CCD area-detector diffractometer
Absorption correction: multi-scan (*SADABS*; Bruker, 2009 ▶) *T*_min_ = 0.362, *T*_max_ = 0.600
25337 measured reflections
7357 independent reflections
5709 reflections with *I* > 2σ(*I*)
*R*_int_ = 0.052

#### Refinement

*R*[*F* ^2^ > 2σ(*F* ^2^)] = 0.042
*wR*(*F* ^2^) = 0.120
*S* = 1.02
7357 reflections
339 parameters
H atoms treated by a mixture of independent and constrained refinement
Δρ_max_ = 1.27 e Å^−3^
Δρ_min_ = −0.94 e Å^−3^

### 

Data collection: *APEX2* (Bruker, 2009 ▶); cell refinement: *SAINT* (Bruker, 2009 ▶); data reduction: *SAINT*; program(s) used to solve structure: *SHELXTL* (Sheldrick, 2008 ▶); program(s) used to refine structure: *SHELXTL*; molecular graphics: *SHELXTL*; software used to prepare material for publication: *SHELXTL* and *PLATON* (Spek, 2009 ▶).

## Supplementary Material

Crystal structure: contains datablocks global, I. DOI: 10.1107/S1600536810051585/sj5073sup1.cif

Structure factors: contains datablocks I. DOI: 10.1107/S1600536810051585/sj5073Isup2.hkl

Additional supplementary materials:  crystallographic information; 3D view; checkCIF report

## Figures and Tables

**Table 1:** Hydrogen-bond geometry (Å, °)

*D*—H⋯*A*	*D*—H	H⋯*A*	*D*⋯*A*	*D*—H⋯*A*
O3—H1*O*3⋯N2^i^	0.83 (3)	2.02 (3)	2.773 (2)	151 (3)
C11—H11*B*⋯O3^i^	0.97	2.39	3.288 (3)	153
C17—H17*A*⋯O3^i^	0.93	2.33	3.203 (3)	157

Symmetry code: (i) 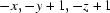.

**Fig. 1.**
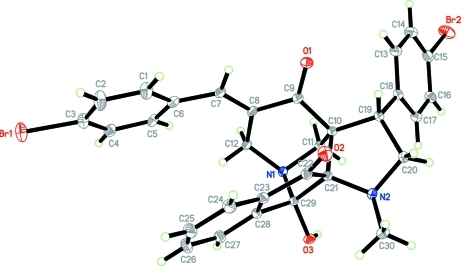
The molecular structure of the title compound, showing 30 % probability displacement ellipsoids for non-H atoms and the atom-numbering scheme.

**Fig. 2.**
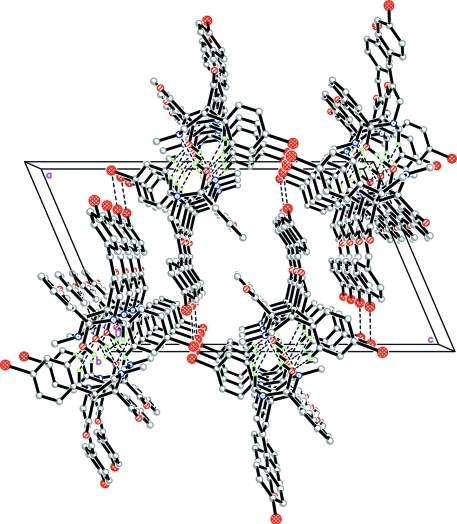
The crystal structure of the title compound, viewed down the b axis, showing the molecules being interconnected into two double-dimeric columns. H atoms not involved in intermolecular interactions (dashed lines) have been omitted for clarity.

## supplementary crystallographic information

### Comment

Highly functionalized pyrrolidines have gained much interest in the past few years as they constitute the main structural element of many natural and synthetic pharmacologically active compounds (Waldmann, 1995). Optically active pyrrolidines have been used as intermediates, chiral ligands or auxiliaries in controlled asymmetric synthesis (Suzuki *et al.*, 1994; Huryn *et al.*, 1991). In view of this importance, the crystal structure of the title compound has been carried out and the results are presented here.

The molecular structure of the title pyrrolidine compound is shown in Fig. 1. The two fused pyrrolidine rings with atom sequences (C10/C11/N1/C21/C29) & (C10/C19/C20/N2/C21) adopt envolope and twisted conformations, respectively; the puckering parameters are Q = 0.454 (2) Å, φ = 37.4 (3)° and Q = 0.338 (2) Å, φ = 334.7 (4)°, respectively (Cremer & Pople, 1975). The piperidine ring (C8/C9/C10/C11/N1/C12) adopts an envelope conformation, with the flap atom (C11) deviating from the mean plane through the remaining five atoms by 0.800 (2) Å; the puckering parameters are Q = 0.622 (2) Å, θ = 141.18 (18)° and φ = 240.6 (3)° (Cremer & Pople, 1975). The 2,3-dihydro-1*H*-inden-1-one moiety (C21-C29/O2) is essentially planar, with a maximum deviation of -0.0163 (19) Å at atom C21. The two bromo-substituted benzene rings (C1-C6 & C13-C18) are inclined at interplanar angles of 14.29 (9) and 61.07 (9)°, respectively, with the 2,3-dihydro-1*H*-inden-1-one moiety. All geometrical parameters are consistent to those observed in closely related structures (Kumar *et al.*, 2010**a*,*b*,c*).

In the crystal structure (Fig. 2), intermolecular O3—H1O3···N2, C11—H11B···O3 and C17—H17A···O3 hydrogen bonds (Table 1) link inversion-related molecules into dimers. An interesting feature of the crystal structure is the short intermolecular Br1···Br2 interaction [3.5140 (6) Å], which is shorter than the sum of the van der Waals radius of bromine atom (3.70 Å). These Br1···Br2 interactions further interconnect these dimers into double-dimeric columns along the *b* axis.

### Experimental

The title compound was synthesized according to a previously described procedure (Kumar *et al.*, 2010**a*,*b*,c*), and was recrystallized from ethyl acetate to afford pale yellow single crystals.

### Refinement

Atom H1O3 was located from a difference Fourier map [O3—H1O3 = 0.82 (3) Å] and allowed to refine freely. The remaining H atoms were placed in their calculated positions, with C—H = 0.93 – 0.97 Å, and refined using a riding model, with *U*_iso_ = 1.2 or 1.5 *U*_eq_(C). A rotating group model was applied to the methyl group.

### Crystal data

**Table:**

C_30_H_24_Br_2_N_2_O_3_	*F*(000) = 1248
*M**_r_* = 620.33	*D*_x_ = 1.633 Mg m^−^^3^
Monoclinic, *P*2_1_/*c*	Mo *K*α radiation, λ = 0.71073 Å
Hall symbol: -P 2ybc	Cell parameters from 7088 reflections
*a* = 13.3490 (18) Å	θ = 2.7–29.9°
*b* = 9.1243 (12) Å	µ = 3.25 mm^−^^1^
*c* = 22.541 (3) Å	*T* = 100 K
β = 113.191 (6)°	Block, yellow
*V* = 2523.7 (6) Å^3^	0.39 × 0.38 × 0.18 mm
*Z* = 4	

### Data collection

**Table:**

Bruker APEXII DUO CCD area-detector diffractometer	7357 independent reflections
Radiation source: fine-focus sealed tube	5709 reflections with *I* > 2σ(*I*)
graphite	*R*_int_ = 0.052
φ and ω scans	θ_max_ = 30.1°, θ_min_ = 2.0°
Absorption correction: multi-scan (*SADABS*; Bruker, 2009)	*h* = −18→18
*T*_min_ = 0.362, *T*_max_ = 0.600	*k* = −12→12
25337 measured reflections	*l* = −31→31

### Refinement

**Table:**

Refinement on *F*^2^	Primary atom site location: structure-invariant direct methods
Least-squares matrix: full	Secondary atom site location: difference Fourier map
*R*[*F*^2^ > 2σ(*F*^2^)] = 0.042	Hydrogen site location: inferred from neighbouring sites
*wR*(*F*^2^) = 0.120	H atoms treated by a mixture of independent and constrained refinement
*S* = 1.02	*w* = 1/[σ^2^(*F*_o_^2^) + (0.067*P*)^2^ + 0.5579*P*] where *P* = (*F*_o_^2^ + 2*F*_c_^2^)/3
7357 reflections	(Δ/σ)_max_ = 0.001
339 parameters	Δρ_max_ = 1.27 e Å^−^^3^
0 restraints	Δρ_min_ = −0.94 e Å^−^^3^

### Special details

**Table:**

Experimental. The crystal was placed in the cold stream of an Oxford Cryosystems Cobra open-flow nitrogen cryostat (Cosier & Glazer, 1986) operating at 100.0 (1)K.
Geometry. All esds (except the esd in the dihedral angle between two l.s. planes) are estimated using the full covariance matrix. The cell esds are taken into account individually in the estimation of esds in distances, angles and torsion angles; correlations between esds in cell parameters are only used when they are defined by crystal symmetry. An approximate (isotropic) treatment of cell esds is used for estimating esds involving l.s. planes.
Refinement. Refinement of F^2^ against ALL reflections. The weighted R-factor wR and goodness of fit S are based on F^2^, conventional R-factors R are based on F, with F set to zero for negative F^2^. The threshold expression of F^2^ > 2sigma(F^2^) is used only for calculating R-factors(gt) etc. and is not relevant to the choice of reflections for refinement. R-factors based on F^2^ are statistically about twice as large as those based on F, and R- factors based on ALL data will be even larger.

### Fractional atomic coordinates and isotropic or equivalent isotropic displacement parameters (Å^2^)

**Table:**

	*x*	*y*	*z*	*U*_iso_*/*U*_eq_	
Br1	0.75505 (2)	−0.12072 (3)	0.698221 (14)	0.03673 (9)	
Br2	0.04211 (2)	1.10857 (3)	0.764653 (13)	0.04012 (10)	
O1	0.42011 (12)	0.74248 (18)	0.64632 (8)	0.0298 (4)	
O2	0.33814 (13)	0.67728 (18)	0.48068 (8)	0.0279 (3)	
O3	0.07211 (11)	0.37326 (16)	0.49214 (7)	0.0203 (3)	
N1	0.19684 (13)	0.41022 (19)	0.59993 (8)	0.0185 (3)	
N2	0.10821 (13)	0.66492 (19)	0.47739 (8)	0.0188 (3)	
C1	0.65306 (19)	0.3089 (3)	0.65943 (12)	0.0309 (5)	
H1A	0.6778	0.3929	0.6461	0.037*	
C2	0.7103 (2)	0.1791 (3)	0.66670 (14)	0.0369 (6)	
H2A	0.7731	0.1759	0.6584	0.044*	
C3	0.67343 (19)	0.0541 (3)	0.68643 (11)	0.0278 (5)	
C4	0.58098 (17)	0.0572 (3)	0.70013 (10)	0.0258 (4)	
H4A	0.5567	−0.0275	0.7132	0.031*	
C5	0.52520 (16)	0.1883 (3)	0.69400 (10)	0.0245 (4)	
H5A	0.4648	0.1918	0.7048	0.029*	
C6	0.55817 (16)	0.3155 (2)	0.67190 (10)	0.0223 (4)	
C7	0.50039 (16)	0.4559 (2)	0.65988 (10)	0.0212 (4)	
H7A	0.5431	0.5379	0.6620	0.025*	
C8	0.39434 (15)	0.4838 (2)	0.64618 (9)	0.0197 (4)	
C9	0.35805 (16)	0.6388 (2)	0.62815 (10)	0.0206 (4)	
C10	0.23733 (15)	0.6582 (2)	0.58527 (9)	0.0178 (4)	
C11	0.17469 (16)	0.5606 (2)	0.61558 (10)	0.0193 (4)	
H11A	0.2017	0.5751	0.6619	0.023*	
H11B	0.0972	0.5818	0.5967	0.023*	
C12	0.30882 (16)	0.3701 (2)	0.64265 (10)	0.0201 (4)	
H12A	0.3273	0.2782	0.6278	0.024*	
H12B	0.3114	0.3541	0.6858	0.024*	
C13	0.23320 (18)	0.9689 (3)	0.66853 (11)	0.0285 (5)	
H13A	0.3058	0.9774	0.6741	0.034*	
C14	0.1991 (2)	1.0335 (3)	0.71292 (12)	0.0321 (5)	
H14A	0.2480	1.0853	0.7478	0.038*	
C15	0.09096 (19)	1.0199 (3)	0.70461 (11)	0.0274 (4)	
C16	0.01713 (19)	0.9433 (2)	0.65255 (11)	0.0273 (4)	
H16A	−0.0553	0.9346	0.6474	0.033*	
C17	0.05257 (18)	0.8798 (2)	0.60831 (11)	0.0247 (4)	
H17A	0.0033	0.8288	0.5732	0.030*	
C18	0.16138 (18)	0.8914 (2)	0.61564 (11)	0.0220 (4)	
C19	0.20385 (16)	0.8202 (2)	0.56940 (10)	0.0202 (4)	
H19A	0.2696	0.8740	0.5731	0.024*	
C20	0.12693 (17)	0.8198 (2)	0.49721 (10)	0.0221 (4)	
H20A	0.0586	0.8675	0.4910	0.027*	
H20B	0.1602	0.8713	0.4720	0.027*	
C21	0.20367 (15)	0.5807 (2)	0.51872 (9)	0.0175 (4)	
C22	0.29550 (16)	0.5715 (2)	0.49399 (10)	0.0207 (4)	
C23	0.32090 (16)	0.4148 (2)	0.49005 (10)	0.0214 (4)	
C24	0.39758 (18)	0.3521 (3)	0.46964 (12)	0.0276 (5)	
H24A	0.4416	0.4107	0.4561	0.033*	
C25	0.40643 (19)	0.2012 (3)	0.47010 (13)	0.0330 (5)	
H25A	0.4578	0.1577	0.4574	0.040*	
C26	0.3394 (2)	0.1134 (3)	0.48941 (13)	0.0328 (5)	
H26A	0.3456	0.0120	0.4887	0.039*	
C27	0.26277 (18)	0.1761 (2)	0.50979 (11)	0.0263 (4)	
H27A	0.2180	0.1174	0.5227	0.032*	
C28	0.25500 (16)	0.3277 (2)	0.51039 (10)	0.0203 (4)	
C29	0.17847 (15)	0.4177 (2)	0.53055 (9)	0.0172 (4)	
C30	0.07438 (18)	0.6445 (3)	0.40754 (10)	0.0245 (4)	
H30A	0.0698	0.5417	0.3978	0.037*	
H30B	0.1268	0.6894	0.3939	0.037*	
H30C	0.0044	0.6891	0.3852	0.037*	
H1O3	0.032 (2)	0.380 (3)	0.5121 (14)	0.024 (7)*	

### Atomic displacement parameters (Å^2^)

**Table:**

	*U*^11^	*U*^22^	*U*^33^	*U*^12^	*U*^13^	*U*^23^
Br1	0.04380 (16)	0.02974 (15)	0.04459 (16)	0.01490 (10)	0.02593 (13)	0.01142 (10)
Br2	0.05369 (18)	0.03787 (16)	0.03862 (15)	−0.01126 (11)	0.02871 (13)	−0.01324 (11)
O1	0.0211 (7)	0.0207 (8)	0.0386 (9)	−0.0050 (6)	0.0022 (7)	−0.0016 (7)
O2	0.0250 (7)	0.0239 (8)	0.0379 (9)	−0.0036 (6)	0.0156 (7)	0.0014 (7)
O3	0.0131 (6)	0.0238 (8)	0.0214 (7)	−0.0039 (5)	0.0041 (6)	−0.0033 (5)
N1	0.0160 (7)	0.0167 (8)	0.0204 (8)	0.0008 (6)	0.0047 (6)	0.0014 (6)
N2	0.0178 (7)	0.0175 (8)	0.0186 (7)	0.0017 (6)	0.0046 (6)	0.0019 (6)
C1	0.0300 (11)	0.0273 (12)	0.0418 (13)	0.0038 (9)	0.0212 (10)	0.0098 (10)
C2	0.0363 (13)	0.0328 (13)	0.0538 (16)	0.0098 (11)	0.0307 (12)	0.0144 (12)
C3	0.0311 (11)	0.0256 (11)	0.0295 (11)	0.0080 (9)	0.0151 (9)	0.0043 (9)
C4	0.0231 (10)	0.0265 (11)	0.0246 (10)	0.0002 (8)	0.0061 (8)	0.0073 (8)
C5	0.0168 (9)	0.0305 (12)	0.0233 (10)	0.0025 (8)	0.0049 (8)	0.0062 (8)
C6	0.0178 (9)	0.0269 (11)	0.0200 (9)	0.0003 (8)	0.0049 (7)	0.0018 (8)
C7	0.0173 (8)	0.0210 (10)	0.0208 (9)	−0.0015 (7)	0.0027 (7)	0.0011 (7)
C8	0.0156 (8)	0.0217 (10)	0.0171 (8)	0.0003 (7)	0.0014 (7)	0.0019 (7)
C9	0.0168 (8)	0.0205 (10)	0.0217 (9)	−0.0009 (7)	0.0044 (7)	−0.0007 (7)
C10	0.0145 (8)	0.0155 (9)	0.0201 (8)	0.0000 (7)	0.0034 (7)	−0.0001 (7)
C11	0.0189 (9)	0.0169 (9)	0.0201 (9)	−0.0003 (7)	0.0057 (7)	−0.0003 (7)
C12	0.0162 (8)	0.0187 (10)	0.0216 (9)	0.0002 (7)	0.0031 (7)	0.0025 (7)
C13	0.0224 (10)	0.0241 (11)	0.0333 (11)	−0.0013 (8)	0.0048 (9)	−0.0068 (9)
C14	0.0321 (11)	0.0281 (12)	0.0311 (11)	−0.0023 (10)	0.0072 (10)	−0.0085 (10)
C15	0.0344 (11)	0.0207 (10)	0.0276 (10)	0.0015 (9)	0.0127 (9)	−0.0006 (8)
C16	0.0286 (10)	0.0215 (11)	0.0329 (11)	−0.0021 (9)	0.0134 (9)	−0.0023 (9)
C17	0.0229 (10)	0.0208 (10)	0.0268 (10)	−0.0020 (8)	0.0058 (8)	−0.0044 (8)
C18	0.0224 (9)	0.0157 (9)	0.0247 (10)	0.0012 (7)	0.0058 (8)	−0.0001 (7)
C19	0.0199 (9)	0.0151 (9)	0.0225 (9)	0.0006 (7)	0.0051 (7)	−0.0002 (7)
C20	0.0243 (10)	0.0166 (9)	0.0220 (9)	0.0015 (8)	0.0053 (8)	0.0020 (7)
C21	0.0144 (8)	0.0167 (9)	0.0197 (9)	0.0005 (7)	0.0050 (7)	0.0002 (7)
C22	0.0166 (8)	0.0229 (10)	0.0219 (9)	−0.0009 (7)	0.0067 (7)	−0.0005 (8)
C23	0.0161 (8)	0.0226 (10)	0.0237 (9)	−0.0015 (7)	0.0061 (8)	−0.0032 (8)
C24	0.0203 (10)	0.0299 (12)	0.0340 (11)	−0.0005 (9)	0.0121 (9)	−0.0052 (9)
C25	0.0241 (10)	0.0334 (13)	0.0419 (13)	0.0034 (9)	0.0137 (10)	−0.0103 (11)
C26	0.0307 (11)	0.0217 (11)	0.0452 (14)	0.0045 (9)	0.0139 (11)	−0.0057 (10)
C27	0.0218 (9)	0.0207 (10)	0.0340 (11)	0.0002 (8)	0.0084 (9)	−0.0026 (9)
C28	0.0164 (8)	0.0192 (10)	0.0228 (9)	0.0010 (7)	0.0051 (7)	−0.0029 (8)
C29	0.0137 (8)	0.0158 (9)	0.0197 (9)	−0.0013 (7)	0.0039 (7)	−0.0008 (7)
C30	0.0222 (9)	0.0282 (11)	0.0204 (9)	−0.0018 (8)	0.0056 (8)	0.0012 (8)

### Geometric parameters (Å, °)

**Table:**

Br1—C3	1.891 (2)	C12—H12B	0.9700
Br2—C15	1.899 (2)	C13—C14	1.384 (3)
O1—C9	1.217 (3)	C13—C18	1.393 (3)
O2—C22	1.217 (3)	C13—H13A	0.9300
O3—C29	1.400 (2)	C14—C15	1.386 (3)
O3—H1O3	0.82 (3)	C14—H14A	0.9300
N1—C12	1.470 (2)	C15—C16	1.388 (3)
N1—C11	1.476 (3)	C16—C17	1.387 (3)
N1—C29	1.486 (3)	C16—H16A	0.9300
N2—C21	1.466 (2)	C17—C18	1.400 (3)
N2—C30	1.469 (3)	C17—H17A	0.9300
N2—C20	1.473 (3)	C18—C19	1.515 (3)
C1—C2	1.384 (3)	C19—C20	1.545 (3)
C1—C6	1.403 (3)	C19—H19A	0.9800
C1—H1A	0.9300	C20—H20A	0.9700
C2—C3	1.382 (3)	C20—H20B	0.9700
C2—H2A	0.9300	C21—C22	1.536 (3)
C3—C4	1.385 (3)	C21—C29	1.571 (3)
C4—C5	1.387 (3)	C22—C23	1.480 (3)
C4—H4A	0.9300	C23—C28	1.391 (3)
C5—C6	1.400 (3)	C23—C24	1.399 (3)
C5—H5A	0.9300	C24—C25	1.382 (4)
C6—C7	1.465 (3)	C24—H24A	0.9300
C7—C8	1.349 (3)	C25—C26	1.392 (4)
C7—H7A	0.9300	C25—H25A	0.9300
C8—C9	1.498 (3)	C26—C27	1.398 (3)
C8—C12	1.522 (3)	C26—H26A	0.9300
C9—C10	1.528 (3)	C27—C28	1.387 (3)
C10—C19	1.545 (3)	C27—H27A	0.9300
C10—C11	1.552 (3)	C28—C29	1.513 (3)
C10—C21	1.556 (3)	C30—H30A	0.9600
C11—H11A	0.9700	C30—H30B	0.9600
C11—H11B	0.9700	C30—H30C	0.9600
C12—H12A	0.9700		
			
C29—O3—H1O3	110.9 (19)	C17—C16—C15	119.2 (2)
C12—N1—C11	108.83 (16)	C17—C16—H16A	120.4
C12—N1—C29	113.86 (16)	C15—C16—H16A	120.4
C11—N1—C29	104.09 (15)	C16—C17—C18	121.0 (2)
C21—N2—C30	116.00 (16)	C16—C17—H17A	119.5
C21—N2—C20	108.01 (15)	C18—C17—H17A	119.5
C30—N2—C20	112.97 (16)	C13—C18—C17	118.1 (2)
C2—C1—C6	120.9 (2)	C13—C18—C19	119.26 (19)
C2—C1—H1A	119.6	C17—C18—C19	122.58 (19)
C6—C1—H1A	119.6	C18—C19—C20	116.89 (17)
C3—C2—C1	119.6 (2)	C18—C19—C10	113.80 (17)
C3—C2—H2A	120.2	C20—C19—C10	104.67 (16)
C1—C2—H2A	120.2	C18—C19—H19A	107.0
C2—C3—C4	121.1 (2)	C20—C19—H19A	107.0
C2—C3—Br1	118.87 (17)	C10—C19—H19A	107.0
C4—C3—Br1	120.01 (18)	N2—C20—C19	106.54 (16)
C3—C4—C5	119.0 (2)	N2—C20—H20A	110.4
C3—C4—H4A	120.5	C19—C20—H20A	110.4
C5—C4—H4A	120.5	N2—C20—H20B	110.4
C4—C5—C6	121.2 (2)	C19—C20—H20B	110.4
C4—C5—H5A	119.4	H20A—C20—H20B	108.6
C6—C5—H5A	119.4	N2—C21—C22	114.61 (16)
C5—C6—C1	118.1 (2)	N2—C21—C10	102.89 (15)
C5—C6—C7	125.05 (19)	C22—C21—C10	113.96 (16)
C1—C6—C7	116.9 (2)	N2—C21—C29	114.45 (15)
C8—C7—C6	129.5 (2)	C22—C21—C29	105.35 (16)
C8—C7—H7A	115.3	C10—C21—C29	105.46 (15)
C6—C7—H7A	115.3	O2—C22—C23	127.68 (19)
C7—C8—C9	115.98 (18)	O2—C22—C21	124.3 (2)
C7—C8—C12	125.56 (19)	C23—C22—C21	108.00 (17)
C9—C8—C12	118.28 (17)	C28—C23—C24	121.0 (2)
O1—C9—C8	122.53 (18)	C28—C23—C22	110.04 (18)
O1—C9—C10	122.10 (19)	C24—C23—C22	129.0 (2)
C8—C9—C10	115.37 (17)	C25—C24—C23	118.4 (2)
C9—C10—C19	113.19 (16)	C25—C24—H24A	120.8
C9—C10—C11	105.79 (16)	C23—C24—H24A	120.8
C19—C10—C11	119.58 (16)	C24—C25—C26	120.8 (2)
C9—C10—C21	112.92 (16)	C24—C25—H25A	119.6
C19—C10—C21	105.32 (16)	C26—C25—H25A	119.6
C11—C10—C21	99.37 (15)	C25—C26—C27	120.7 (2)
N1—C11—C10	103.57 (15)	C25—C26—H26A	119.6
N1—C11—H11A	111.0	C27—C26—H26A	119.6
C10—C11—H11A	111.0	C28—C27—C26	118.6 (2)
N1—C11—H11B	111.0	C28—C27—H27A	120.7
C10—C11—H11B	111.0	C26—C27—H27A	120.7
H11A—C11—H11B	109.0	C27—C28—C23	120.47 (19)
N1—C12—C8	114.38 (16)	C27—C28—C29	127.28 (19)
N1—C12—H12A	108.7	C23—C28—C29	112.24 (18)
C8—C12—H12A	108.7	O3—C29—N1	110.15 (16)
N1—C12—H12B	108.7	O3—C29—C28	107.30 (16)
C8—C12—H12B	108.7	N1—C29—C28	116.11 (16)
H12A—C12—H12B	107.6	O3—C29—C21	113.68 (16)
C14—C13—C18	121.6 (2)	N1—C29—C21	105.32 (15)
C14—C13—H13A	119.2	C28—C29—C21	104.35 (16)
C18—C13—H13A	119.2	N2—C30—H30A	109.5
C13—C14—C15	119.1 (2)	N2—C30—H30B	109.5
C13—C14—H14A	120.5	H30A—C30—H30B	109.5
C15—C14—H14A	120.5	N2—C30—H30C	109.5
C14—C15—C16	121.0 (2)	H30A—C30—H30C	109.5
C14—C15—Br2	119.81 (18)	H30B—C30—H30C	109.5
C16—C15—Br2	119.24 (17)		
			
C6—C1—C2—C3	0.1 (4)	C30—N2—C21—C22	40.0 (2)
C1—C2—C3—C4	−1.1 (4)	C20—N2—C21—C22	−88.0 (2)
C1—C2—C3—Br1	−178.4 (2)	C30—N2—C21—C10	164.25 (16)
C2—C3—C4—C5	−0.2 (4)	C20—N2—C21—C10	36.29 (19)
Br1—C3—C4—C5	177.03 (17)	C30—N2—C21—C29	−81.9 (2)
C3—C4—C5—C6	2.5 (3)	C20—N2—C21—C29	150.15 (17)
C4—C5—C6—C1	−3.5 (3)	C9—C10—C21—N2	−154.75 (16)
C4—C5—C6—C7	175.8 (2)	C19—C10—C21—N2	−30.76 (19)
C2—C1—C6—C5	2.1 (4)	C11—C10—C21—N2	93.58 (16)
C2—C1—C6—C7	−177.2 (2)	C9—C10—C21—C22	−30.1 (2)
C5—C6—C7—C8	−24.2 (4)	C19—C10—C21—C22	93.93 (19)
C1—C6—C7—C8	155.0 (2)	C11—C10—C21—C22	−141.74 (17)
C6—C7—C8—C9	−172.5 (2)	C9—C10—C21—C29	84.99 (19)
C6—C7—C8—C12	2.6 (4)	C19—C10—C21—C29	−151.01 (15)
C7—C8—C9—O1	−26.2 (3)	C11—C10—C21—C29	−26.68 (17)
C12—C8—C9—O1	158.3 (2)	N2—C21—C22—O2	54.7 (3)
C7—C8—C9—C10	153.83 (18)	C10—C21—C22—O2	−63.4 (3)
C12—C8—C9—C10	−21.6 (3)	C29—C21—C22—O2	−178.56 (19)
O1—C9—C10—C19	−1.2 (3)	N2—C21—C22—C23	−125.52 (18)
C8—C9—C10—C19	178.73 (17)	C10—C21—C22—C23	116.32 (19)
O1—C9—C10—C11	−134.0 (2)	C29—C21—C22—C23	1.2 (2)
C8—C9—C10—C11	45.9 (2)	O2—C22—C23—C28	179.2 (2)
O1—C9—C10—C21	118.4 (2)	C21—C22—C23—C28	−0.6 (2)
C8—C9—C10—C21	−61.7 (2)	O2—C22—C23—C24	−0.9 (4)
C12—N1—C11—C10	75.54 (18)	C21—C22—C23—C24	179.3 (2)
C29—N1—C11—C10	−46.21 (18)	C28—C23—C24—C25	0.0 (3)
C9—C10—C11—N1	−72.53 (18)	C22—C23—C24—C25	−179.9 (2)
C19—C10—C11—N1	158.33 (16)	C23—C24—C25—C26	1.0 (4)
C21—C10—C11—N1	44.65 (17)	C24—C25—C26—C27	−1.1 (4)
C11—N1—C12—C8	−49.2 (2)	C25—C26—C27—C28	0.0 (4)
C29—N1—C12—C8	66.4 (2)	C26—C27—C28—C23	1.1 (3)
C7—C8—C12—N1	−153.2 (2)	C26—C27—C28—C29	−179.9 (2)
C9—C8—C12—N1	21.8 (3)	C24—C23—C28—C27	−1.1 (3)
C18—C13—C14—C15	0.4 (4)	C22—C23—C28—C27	178.8 (2)
C13—C14—C15—C16	−0.3 (4)	C24—C23—C28—C29	179.77 (19)
C13—C14—C15—Br2	−179.64 (18)	C22—C23—C28—C29	−0.3 (2)
C14—C15—C16—C17	−0.1 (4)	C12—N1—C29—O3	146.41 (17)
Br2—C15—C16—C17	179.31 (17)	C11—N1—C29—O3	−95.24 (18)
C15—C16—C17—C18	0.3 (3)	C12—N1—C29—C28	24.2 (2)
C14—C13—C18—C17	−0.1 (3)	C11—N1—C29—C28	142.58 (17)
C14—C13—C18—C19	−178.7 (2)	C12—N1—C29—C21	−90.63 (18)
C16—C17—C18—C13	−0.2 (3)	C11—N1—C29—C21	27.72 (18)
C16—C17—C18—C19	178.3 (2)	C27—C28—C29—O3	−57.1 (3)
C13—C18—C19—C20	−144.0 (2)	C23—C28—C29—O3	121.97 (18)
C17—C18—C19—C20	37.5 (3)	C27—C28—C29—N1	66.6 (3)
C13—C18—C19—C10	93.7 (2)	C23—C28—C29—N1	−114.36 (19)
C17—C18—C19—C10	−84.8 (2)	C27—C28—C29—C21	−178.0 (2)
C9—C10—C19—C18	−92.9 (2)	C23—C28—C29—C21	1.0 (2)
C11—C10—C19—C18	32.8 (2)	N2—C21—C29—O3	8.9 (2)
C21—C10—C19—C18	143.27 (17)	C22—C21—C29—O3	−117.90 (17)
C9—C10—C19—C20	138.31 (18)	C10—C21—C29—O3	121.25 (17)
C11—C10—C19—C20	−96.0 (2)	N2—C21—C29—N1	−111.75 (17)
C21—C10—C19—C20	14.5 (2)	C22—C21—C29—N1	121.43 (16)
C21—N2—C20—C19	−27.7 (2)	C10—C21—C29—N1	0.58 (18)
C30—N2—C20—C19	−157.37 (17)	N2—C21—C29—C28	125.49 (17)
C18—C19—C20—N2	−119.92 (19)	C22—C21—C29—C28	−1.32 (19)
C10—C19—C20—N2	7.0 (2)	C10—C21—C29—C28	−122.17 (16)

### Hydrogen-bond geometry (Å, °)

**Table:**

*D*—H···*A*	*D*—H	H···*A*	*D*···*A*	*D*—H···*A*
O3—H1O3···N2^i^	0.83 (3)	2.02 (3)	2.773 (2)	151 (3)
C11—H11B···O3^i^	0.97	2.39	3.288 (3)	153
C17—H17A···O3^i^	0.93	2.33	3.203 (3)	157

Symmetry codes: (i) −*x*, −*y*+1, −*z*+1.
